# Hospitalisations and Costs of Chronic Health Conditions Among Long‐Term Survivors of Childhood, Adolescent, and Young Adult Cancers in Queensland, Australia

**DOI:** 10.1002/pon.70395

**Published:** 2026-02-07

**Authors:** Doreen Nabukalu, Louisa G. Collins, Daniel Lindsay, John Lowe, Katharina M. D. Merollini

**Affiliations:** ^1^ School of Health University of the Sunshine Coast Sunshine Coast Australia; ^2^ Viertel Cancer Research Centre Cancer Council Queensland Fortitude Valley Australia; ^3^ Population Health Program QIMR Berghofer Medical Research Institute Herston Australia; ^4^ Faculty of Health Medicine and Behavioural Sciences ‐ The University of Queensland Herston Australia; ^5^ Cancer and Palliative Care Outcomes Centre and School of Nursing Queensland University of Technology Brisbane Australia

**Keywords:** adolescent, cancer, cancer survivors, child, chronic disease, health care costs, neoplasms, oncology, paediatrics, young adult

## Abstract

**Background:**

Adult cancer survivors are likely to be hospitalised with chronic illnesses, although evidence for childhood and AYA survivors is limited.

**Aim:**

This study quantified hospitalisations and the costs of health services used by survivors of childhood, adolescent, and young adult (AYA) cancers with and without chronic conditions.

**Methods:**

We assessed long‐term survivors (≥ 5 years past diagnosis) of childhood and AYA cancers diagnosed at ages 0–39 years between 1997 and 2011 in Queensland, Australia. Utilising a linked administrative dataset, we determined the prevalence of chronic conditions from hospital records using classification codes (ICD‐10‐AM) and quantified hospitalisations and associated costs in 2024 Australian dollars (AU$). Generalised linear regression modelling was used to examine how chronic conditions affected healthcare costs, controlling for clinical and socio‐demographic factors.

**Results:**

Of 14,422 participants, 16% (*n* = 2286) were hospitalised with at least one chronic disease, with hypertension (*n* = 675, 4.7%) and depression (*n* = 463, 3.2%) being the most common. Inpatient admissions were significantly higher for survivors with chronic conditions (mean 3, SD = 10) compared to those without chronic conditions (mean 1, SD = 4). The mean annual costs were highest for those with chronic kidney disease (AU$26,428, SD = AU$30,331), schizophrenia (AU$22,835, SD = AU$37,204), epilepsy (AU$22,361, SD = AU$37,224), paralysis (AU$22,051, SD = AU$32,165) and chronic heart failure (AU$21,912 SD = AU$38,763). Hypertension (AU$5.4 million) and depression (AU$4.3 million) incurred the highest total costs over the follow‐up period.

**Conclusion:**

Implementing targeted survivorship care and preventative measures for high‐cost conditions such as schizophrenia and chronic kidney disease may optimise healthcare resource use and reduce the economic burden for this population.

## 1 Background

Chronic health conditions are Australia's largest disease burden comprising 91% of the non‐fatal burden, 78% of the fatal burden, and over one‐third of the total healthcare expenditure [1, 2, 3]. They encompass a range of long‐term illnesses, which last at least 6 months, and gradually lower the quality of life [4]. According to the Australian Institute of Health and Welfare (AIHW), close to half (47%) of Australians have at least one chronic health condition [5].

Although prevalence estimates for AYAs (defined in this study as being aged 15–39 years) are not routinely reported as a single group, national data reports around 45% of Australian children (defined in this study as being aged 0–14 years) live with at least one chronic condition [6]. A recent study in NSW highlighted this growing health concern in adolescents particularly in relation to mental health disorders with well‐documented impact on psychological well‐being, and developmental outcomes in young people [7]. Among Australian children, asthma, hay fever, and mental health problems are common, while AYA often experience back problems, asthma, arthritis, and diabetes [8].

Children and AYA with a previous cancer diagnosis are at a higher risk of developing a chronic condition such as cardiomyopathy, stroke, chronic kidney disease, asthma, depression, hypertension, and arthritis among others, compared to their counterparts without a history of cancer [9, 10, 11, 12, 13]. As reported in international studies with large survivorship cohorts, about 60%–88% of childhood and AYA cancer survivors have at least one chronic health condition, with an estimated 25%–40% developing severe, disabling, or life‐threatening conditions over the life course [11, 12, 13]. This heightened risk is attributed to the effects of cancer treatment, including cardiovascular and respiratory complications, hormonal irregularities, and mental health complications, which often emerge and persist years after completion of treatment. Furthermore, the effects of chronic psychological conditions such as depression and schizophrenia extend far beyond medical outcomes and health care costs. They additionally cause disruptions in education, employment, and social participation, which ultimately compound the economic and psychosocial burden experienced by survivors [14, 15, 16].

Although prior studies report a high prevalence of chronic conditions among childhood and AYA cancer survivors, population‐based evidence for long‐term survivors in Australia is limited. While AIHW's National Centre for Monitoring Chronic Conditions (NCMCC), conducts surveillance of chronic health conditions for the general population, it does not capture condition‐specific information for cancer survivor populations. Additionally, although chronic conditions require ongoing medical attention due to their persistent nature [8, 17], there is limited evidence quantifying their impact on health service use, such as hospitalisation and the economic implications in high‐risk groups including survivors of childhood and AYA cancer [17].

To address these gaps, our study used population‐based linked administrative data from Queensland to examine the chronic diseases in admitted long‐term childhood and AYA cancer survivors. We describe and quantify hospitalisations and the costs of all health services used, comparing cancer survivors hospitalised with a chronic condition as a principle or other diagnosis and those without chronic conditions. Highlighting the concentration of the health care cost in young cancer survivors is critical for understanding the broader impact of cancer survivorship on the demand of health services [18] and for informing targeted allocation of resources to survivor populations with high health care needs.

## 2 Methods

### 2.1 Study Design

We undertook a cost analysis based on a retrospective longitudinal cohort study with linked administrative healthcare data [19]. We conducted the analysis from the government health provider's perspective which included public hospital and medical service costs and excluded patient out‐of‐pocket expenses and private health insurance payments.

### 2.2 Australia's Healthcare System

Australia has a predominantly publicly funded healthcare system, with the Federal Government contributing about two‐thirds of the healthcare budget and the remainder covered by private health insurance and patient out‐of‐pocket expenses [20]. Through Medicare, the national health insurance scheme, eligible residents receive free public hospital services and subsidised nonhospital services via the Medicare Benefits Schedule (MBS). This covers general practitioner visits, specialist consultations, diagnostic imaging, and pathology. The Pharmaceutical Benefits Scheme (PBS) provides subsidised medicines. In Queensland, the Queensland Children's Hospital delivers, comprehensive cancer care for children while the Queensland Youth Cancer Service (QYCS) offers specialised care for AYA (aged 15–25) through a state‐wide network of clinicians across public and private healthcare systems. After treatment, follow up and survivorship care is continued through hospital‐based clinics led by specialists and general practitioners (GPs), who collaborate to manage late effects, comorbidities, and disease surveillance throughout the transition to adult care [21, 22, 23].

### 2.3 Data Sources and Data Linkage

**FIGURE 1:**
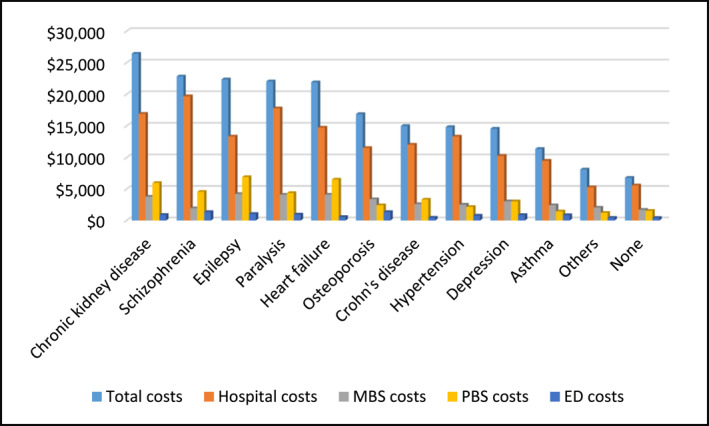
Mean annual healthcare costs by specific chronic condition and health service. MBS, medicare benefits schedule; PBS, pharmaceutical benefits scheme.

We used data from a population‐based data linkage study [19], which comprised all people diagnosed with cancer between 1st January 1997 and 31st December 2015 in Queensland, Australia. Queensland is the third most populous state in Australia, with a population of approximately 5.4 million residents [24]. All cancer diagnoses in the state, except for basal and squamous cell carcinoma of the skin, must be reported to the Queensland Cancer Registry (QCR) with relevant information. Using unique identifiers and probabilistic methods, selected QCR patients were linked to other administrative databases, including the Queensland Hospital Admitted Patient Data Collection (QHAPDC), Emergency Department Information System (EDIS), Medicare Benefits Schedule (MBS) and Pharmaceutical Benefits Scheme (PBS). More information on the data linkage process is available in a previous publication [19]. This linkage process generated a comprehensive dataset with information on the socio‐demographics, clinical data, health service use and costs, as shown in Supporting Information S1: Figure 1.

### 2.4 Study Population and Sample Selection Criteria

The study population comprised individuals diagnosed with cancer in Queensland between January 1997 and December 2011. We included long‐term survivors of childhood and AYA cancer, defined as those aged 0–39 years at diagnosis, and alive at least 5 years after diagnosis. All individuals with multiple primary cancer diagnoses were included; however, baseline demographic characteristics and subsequent analyses were based on the individual's first (and therefore primary) cancer diagnosis. We identified chronic diseases from the International Statistical Classification of Diseases and Related Health Problems, Tenth Revision, Australian Modification (ICD‐10‐AM) codes recorded in the hospital data. In Australia, the ICD‐10‐AM classifies diseases, injuries, and related health problems. In the tenth revision, supplementary codes for 29 clinically significant chronic health conditions and the new Australian coding standard (*0003 Supplementary codes for chronic conditions*) were developed [25]. We used the Independent Health and Aged Care Pricing Authority (IHACPA) mapping algorithms [26] to convert the supplementary codes to their equivalent ICD‐10‐AM codes and code descriptions. For our analysis, we included ICD‐10‐AM codes for a principal or additional diagnosis during an episode of care. Therefore, survivors without hospital data after their cancer diagnosis were excluded (Supporting Information S1: Figure 1).

We obtained ethics approval from the Australian Institute of Health and Welfare (AIHW #EO2017/3/348) and the University of the Sunshine Coast Human Research Ethics Committee (UniSC HREC Approval #A/17/941).

### 2.5 Cancer Site Classifications

We assigned cancer sites using two classification systems. The ICD‐03 for AYA aged 15–39 years [27] or the International Childhood Cancer Classification system (ICCC) for childhood cancers (0–14 years) [28]. In consideration of the different cancer classifications, we summarised cancer types according to these broad groups (children or AYA).

### 2.6 Socio‐Economic Indexes for Areas (SEIFA) and Accessibility/Remoteness Index of Australia (ARIA)

We used the ARIA to assess the geographical distribution of patients [29]. Given the small numbers of cancer survivors residing in more remote areas, we compressed the ARIA categories into ‘major cities’, ‘regional/outer regional’ and ‘remote/very remote’ locations. SEIFA was used to compare outcomes across different socioeconomic groups. SEIFA scores provide a relative measure of socio‐economic advantage and disadvantage. The scores are based on census data on people's access to materials and resources, such as education and employment based on their location of residence [30]. We categorised SEIFA into quintiles, with the first quintile being the least disadvantaged and the fifth quintile being the most advantaged.

### 2.7 Study Outcome Measures

Our primary outcomes were prevalence of chronic conditions, health service use and annual direct healthcare costs. We quantified the inpatient hospital admissions and length of stay using variables in the QHAPDC dataset and valued these admissions using cost weights from the Australian Refined Diagnosis‐Related Groups (AR‐DRG) codes related to each episode of care. Each AR‐DRG code for public hospital admissions and Urgency Related Groups (URG) codes for emergency presentations was selected from the 2018 National Hospital Cost Data Collection (NHCDC) report [31]. We extracted government costs for out‐of‐hospital medical services and pharmaceuticals from linked MBS and PBS records, respectively.

### 2.8 Data Analysis

Descriptive statistics were conducted for baseline factors including age at diagnosis, sex, country of birth, survival status, geographical remoteness, and socio‐economic status. For continuous variables, we reported means and standard deviations (SD), medians and inter‐quartile ranges (IQR), while we described frequencies and percentages for categorical variables. For people with more than one cancer diagnosis, baseline factors were based on the first primary cancer diagnosed.

The frequency of conditions was counted and aggregated per person, and the population was stratified into two groups: having a chronic condition or no chronic condition. Depending on the frequencies in each age group, Chi‐square or Fisher's exact tests were used to compare the prevalence of chronic health conditions among children and AYA.

We assessed outcomes for the long‐term phase of care, which spans from the fifth year after diagnosis to 31st December 2016 or one year before death for deceased participants (Supporting Information S1: Figure 2). This assessment period was used to generate the follow‐up person‐years for each participant, potentially ranging from 0.1 to 15 years.

Counts for inpatient hospital admissions and aggregated days for length of stay were generated per person. These were divided by the person‐years in the assessment period to generate the mean hospital admissions per person per year.

Total healthcare costs were the sum of the costs for hospital admissions, emergency department presentations, medical services, and pharmaceuticals. We divided the total costs by the person years to generate costs per person per year. Means and SDs, and medians and IQRs were calculated for the total healthcare costs and for each type of health service. Using the non‐parametric Mann‐Whitney test, we assessed the differences in hospital admissions, length of stay, and healthcare costs between people with and without chronic health conditions. Only people who used services in the long‐term phase were included in the cost calculations.

We assessed the effect of having a chronic health condition on healthcare costs using a generalised linear regression model with a log link function and gamma distribution. Given that cost data is typically right skewed, we selected this distribution and function because of their optimal model fit diagnostics. The covariates were age at diagnosis, person‐years, number of prior malignancies, country of birth, sex, mortality during follow‐up, ARIA, and SEIFA. The cost ratio (CR) was calculated, and a ratio greater than 1 signifies a higher cost than the referent group.

Cost estimates were calculated in Australian dollars, adjusting for inflation to 2024 using the Consumer Price Index's healthcare component. Statistical significance was determined using a *p*‐value of less than 0.05. All statistical analyses were conducted using STATA software, version 16.

## 3 Results

The final sample size comprised 14,422 participants, representing 57% of the total study sample, as shown in Supporting Information S1: Figure 1.

### 3.1 Study Population Characteristics

Most participants were aged 24–39 years (75.3%) with a mean age of 28.9 years (SD:9.5) at diagnosis and born in Australia (84.9%). Slightly more participants were female (57.4%) and the majority lived in major cities (59.1%). Participants were followed up over a mean of 6.5 (SD:4.3) person‐years and 4.3% (*n* = 624) of participants died during the cohort period. SEIFA quintiles were similar in terms of study participant distribution, varying from 19.3% to 24.2%, except for the least disadvantaged group (quintile 1), which comprised 11.9% of participants. The most common childhood cancers were leukaemias (*n* = 507, 38.7%), lymphomas (*n* = 139, 10.6%), Central nervous system (CNS) cancers (*n* = 114, 8.7%), malignant epithelial neoplasms (*n* = 122, 9.3%), and neuroblastoma and other peripheral nervous cell tumours (*n* = 77, 5.9%). Among AYAs, the predominant cancers were melanoma (*n* = 4336, 33.1%), breast cancer (*n* = 1539, 11.7%), digestive cancers (*n* = 983, 7.5%), thyroid and other endocrine glands (*n* = 1287, 9.8%), and male (*n* = 1,075, 8.2%) and female (*n* = 877, 7.4%) genital organ cancers (Table 1).

**TABLE 1:** Demographic and clinical characteristics of long‐term survivors of childhood and AYA cancers diagnosed with cancer with between 1997 and 2010 in Queensland (*N* = 14,422).

Characteristic	*N*	%
Age at diagnosis		
Mean (SD)	29 (9.5)	
Follow up person years		
Mean (SD)	6.5 (4.3)	
Age categories, years		
0–14	1310	9.1
15–24	2255	15.6
25–39	10857	75.3
Sex		
Male	6123	42.5
Female	8299	57.5
Status (December 31, 2016)		
Alive	13798	95.7
Dead	624	4.3
Country of birth		
Australia	12247	84.9
Others^f^	2175	15.1
Social economic index for areas (SEIFA)		
Quintile 1 (least advantaged)	1713	11.9
Quintile 2	2781	19.3
Quintile 3	3484	24.2
Quintile 4	3378	23.4
Quintile 5 (most advantaged)	2990	20.7
Accessibility/remote index of Australia		
Major cities	8531	59.1
Regional	5492	38.1
Remote	359	2.5
Chronic conditions		
No	12136	84.1
Yes	2286	15.8
Number of chronic conditions		
0	12136	84.1
1	1729	12
2 or more	557	3.9
Five most prevalent childhood cancers.		
Leukaemias^a^	507	38.7
Central nervous system^b^	114	8.7
Lymphomas^c^	139	10.6
Other malignant epithelial neoplasms^d^	122	9.3
Neuroblastoma^e^	77	5.9
Five most prevalent AYA cancers.		
Melanoma	4336	33.1
Breast cancer	1539	11.7
Digestive organs	983	7.5
Thyroid and other endocrine glands.	1287	9.8
Female genital organs	1075	8.2
Number of primary cancer sites		
One	13974	96.9
2 or more cancers	450	3.1

^a^ Leukaemias, myeloproliferative diseases & myelodysplastic diseases.

^b^ CNS & miscellaneous intracranial & intraspinal neoplasms.

^c^ Lymphomas & reticuloendothelial neoplasms.

^d^ Other Malignant epithelial neoplasms & malignant melanomas.

^e^ Neuroblastoma & other peripheral nervous cell tumours.

^f^ Includes other countries besides Australia and whose birthplace wasn't recorded.

### 3.2 Prevalence of Chronic Health Conditions

Approximately 16% of the survivors (*n* = 2286) presented with a chronic condition as a principal or other diagnosis at hospital admissions. These were most prevalent among those aged 25–39 years (*n* = 1733, 75.8%) compared with those aged 15–24 years (*n* = 275, 12.0%) and children 0–14 years old (*n* = 278, 12.2%) (Table 2).

**TABLE 2:** Prevalence of chronic conditions among childhood and AYA cancer survivors by age at diagnosis (*N* = 14,422).

	Total (*N* = 14,422)	0–14 years (*N* = 1310)	15–24 years (*N* = 2255)	25–39 years (*N* = 10,857)	*p*‐value
		*n* (%)	*n* (%)	*n* (%)	
Chronic conditions					
Yes	2286 (15.8)	278 (21.2)	275 (12.2)	1733 (16.0)	
No	12136 (84.1)	1032 (78.8)	1980 (87.8)	9124 (84.0)	
Specific conditions					
Hypertension	675 (4.7)	129 (9.8)	52 (2.3)	494 (4.5)	< 0.001
Depression	463 (3.2)	15 (1.1)	78 (3.5)	370 (3.4)	< 0.001
Arthritis and osteoarthritis	350 (2.4)	07 (0.5)	26 (1.1)	317 (2.9)	< 0.001
Asthma^a^	299 (2.1)	55 (4.2)	48 (2.1)	196 (1.8)	< 0.001
Paralysis	253 (1.7)	34 (2.6)	32 (1.4)	187 (1.7)	0.031
Epilepsy	196 (1.4)	23 (1.8)	27 (1.2)	146 (1.3)	0.368
Chronic heart failure	162 (1.1)	15 (1.1)	23 (1.0)	124 (1.1)	0.878
Ischaemic heart disease	122 (0.8)	00 (0.0)	05 (0.2)	117 (1.0)	< 0.001
Chronic kidney diseases^b^	117 (0.8)	02 (0.1)	19 (0.8)	96 (0.9)	0.007
Schizophrenia	99 (0.7)	01 (0.1)	20 (0.9)	78 (0.7)	0.003
Crohn's disease	72 (0.6)	00 (0,0)	10 (0.4)	62 (0.6)	0.004
Disorder of intellectual development	66 (0.5)	10 (0.8)	14 (0.6)	66 (0.5)	0.074
Bronchiectasis^c^	66 (0.5)	35 (2.7)	08 (0.3)	23 (0.2)	< 0.001
Osteoporosis	26 (0.2)	09 (0.7)	05 (0.2)	12 (0.1)	< 0.001
Systemic lupus erythematosus	20 (0.1)	0 (0.0)	03 (0.1)	17 (0.2)	0.468
Multiple sclerosis	19 (0.1)	0 (0.0)	0 (0.0)	19 (0.2)	0.048
COPD^d^	16 (0.1)	0 (0.0)	01 (0.0)	15 (0.1)	0.375
Cerebral palsy	15 (0.1)	04 (0.3)	05 (0.2)	06 (0.1)	0.005
Chronic respiratory failure	14 (0.1)	02 (0.1)	01 (0.0)	11 (0.1)	0.582
Others^e^	14 (0.1)	0 (0.0)	04 (0.2)	10 (0.1)	

^a^ Asthma without mention of COPD.

^b^ Chronic kidney diseases stage 3–5.

^c^ Bronchiectasis, without mention of cystic fibrosis.

^d^ Chronic obstructive pulmonary disease.

^e^ Others include: Cystic fibrosis, Chronic liver failure, Spina bifida and dementia.

Overall, the most common chronic conditions were hypertension (*n* = 675, 4.7%), depression (*n* = 463, 3.2%), arthritis and osteoarthritis (*n* = 350, 2.4%), asthma (*n* = 299, 2.1%), paralysis (*n* = 253, 1.7%), epilepsy (*n* = 196, 1.4%) and chronic heart failure (*n* = 162, 1.1%). Multi‐morbidity was not common in our study sample with and only occurred in 3.8% of participants (*n* = 557), mostly young adults aged 25 years and above having at least two chronic conditions (Table 2).

### 3.3 All‐Cause Health Service Use

Overall, our sample had one (SD = 4.5) inpatient admission and 2.5 (SD = 8.4) days of stay per person per year (Table 3). Those with a chronic condition had an annual mean of 3 (SD = 10) inpatient admissions and 6.4 (SD = 15.3) days of stay which was significantly higher than those without chronic conditions (mean 1, SD = 4 inpatient admission and mean 1.5, SD = 5.1 days of stay) per person per year (Table 3).

**TABLE 3:** All cause health service use and costs per person per year for long‐term cancer survivors (*N* = 13,743).

Variable	No chronic condition	Chronic condition	Total	*p*‐value
Number of hospital admissions				
*N*	7334	1782	9116	
Mean (SD)	1.0 (4)	3.0 (10)	1.0 (5)	< 0.001
Median (IQR)	0.3 (0.2–0.7)	0.7 (0.4–1.9)	0.4 (0.2–0.8)	
Total	6964	5001	11965	
Length of stay				
*N*	7334	1782	9116	
Mean (SD)	1.5 (5.1)	6.4 (15.3)	2.5 (8.4)	< 0.001
Median (IQR)	0.5 (0.2–1.1)	1.6 (0.6–5.5)	1 (0.2–2.0)	
Total	11186	11385	22571	
Healthcare costs (AU$)				
Total cost				
*N*	11554	2189	13743	
Mean (SD)	$6798 ($19,055)	$19,227 ($36,957)	$8778 ($23,311)	< 0.001
Median (IQR)	$2294 ($970–$2294)	$7496 ($3246–$19,110)	$2721 ($1098–$6700)	
Total	$78,500,000	$42,100,000	$121,000,000	
MBS costs				
*N*	11401	2146	13547	
Mean (SD)	$1697 ($1567)	$3054 ($3914)	$1912 ($2801)	< 0.001
Median (IQR)	$1055 ($561–$1930)	$2002 ($1023–$3651)	$1155 ($599–$2174)	
Total	$19,300,000	$6,600,000	$25,900,000	
PBS costs				
*N*	10661	2098	12759	
Mean (SD)	$1539 ($8861)	$3433 ($9883)	$1850 ($9064)	< 0.001
Median (IQR)	$29 ($0–$180)	$399 ($53–$2235)	$45 ($0–$314)	
Total	$16,400,000	$7,200,000	$23,600,000	
Hospital costs				
*N*	7334	1782	9116	
Mean (SD)	$5569 ($16,490)	$15,049 ($32,688)	$7422 ($21,016)	< 0.001
Median (IQR)	$1849 ($802–$4400)	$5398 ($2178–$5398)	$2226 ($918–$5694)	
Total	$40,800,000	$26,800,000	$67,700,000	
Emergency visits				
*N*	4777	1400	6177	
Mean (SD)	$407 ($830)	$1081 ($2691)	$560 ($1501)	< 0.001
Median (IQR)	$210 ($108–$430)	$459 ($200–$1014)	$244 ($120–$535)	
Total	$1,900,000	$1,500,000	$3,500,000	

*Note:* Only long‐term survivors who used at least one of the health services during the assessment period were included in this analysis. Total Cost estimates have been rounded to the nearest one hundred thousand.

Abbreviations: CI, confidence interval; IQR, interquartile range; MBS, medicare benefits schedule; PBS, pharmaceutical benefits scheme; SD, standard deviation.

### 3.4 Healthcare Costs Among Long‐Term Cancer Survivors

The total healthcare costs for the sample during the survivorship period were AU$121 million (Table 3). By type of health service, hospital admission costs were the highest (AU$67.7 million), followed by costs incurred by the MBS (AU$25.9 million), PBS (AU$23.6 million) and emergency department (AU$3.4 million). Overall, the annual healthcare costs per person per year were mean AU$8778 (SD = AU$23,311) and median AU$2721 (IQR = AU$1098–$6700) during the long‐term survivorship period. The costs were significantly higher for people with chronic conditions (mean AU$19,227, SD = AU$36,957 and median AU$7,496, IQR = AU$3246–$19,110) compared with those without chronic health conditions (mean AU$6,798, SD = AU$19,055 and median AU$2,294, IQR = AU$970–$2294) (Table 3). This pattern was observed for all health services. The highest costs per person per year were related to hospital admissions among those with (mean AU$15,049, SD = $32,688 and median AU$5,398, IQR = AU$2178–$5398) and without chronic conditions (mean AU$5,569, SD = $16,490 and median AU$1,849, IQR AU$802–$4400). Emergency department costs per person per year contributed the least cost for people with chronic condition (mean AU$1,081, SD = AU$2691 and median AU$459, IQR AU$200–$1014) and for those without chronic condition (mean AU$407, SD = AU $830 and median AU$210, IQR = AU$108–$430) (Table 3).

### 3.5 Healthcare Costs by Type of Chronic Health Condition

Annual costs were highest for people with chronic kidney disease (mean AU$26,428, SD = AU$30,331 and median AU$15,888, IQR = AU$5651–$35,414) and schizophrenia (mean AU$22,835 SD = AU$37,204 and median AU$10,460, IQR = AU$4261–$20,924) (Supporting Information S1: Table 1). These were followed by epilepsy (mean AU$22,361, SD = AU$37,224 and median AU$9,889, IQR = AU$4749–$18,325), paralysis (mean AU$22,051, SD = AU$32,165 and median AU$9,285, IQR = AU$4033–$27,679) and chronic heart failure (mean AU$21,912, SD = AU$38,763 and median AU$6,346, IQR = AU$2652–$20,935). However, in terms of cumulative costs, hypertension and depression had the highest contribution to the total healthcare costs of over $5.4 million and $4.3 million, respectively. Irrespective of the condition, hospitalisation was the biggest driver of the total healthcare costs (Figure 1) (Supporting Information S1: Table 1).

### 3.6 Factors Associated With High Healthcare Costs

Having a chronic health condition was associated with 2.3‐fold higher healthcare costs than those without a chronic condition, after accounting for patient and clinical factors (Table 4). Having chronic kidney disease and schizophrenia resulted in the highest costs, with a 3.5‐fold increase and 1.9‐fold increase compared to those without a chronic condition, respectively. The number of previously diagnosed cancers had a 2.2‐fold increase in healthcare costs. Females had 1.3 times higher costs than males. Age at diagnosis is less likely to affect healthcare costs; costs slightly decrease with a unit increase in the follow up person years (Cost ratio = 0.94, *p* < 0.001). Compared to people born in Australia, costs were slightly less for those born outside of Australia (Cost ratio = 0.88, *p* = 0.034). Healthcare costs for deceased participants were 3.7 times higher than for living participants. Geographic location and socioeconomic status had no significant association with healthcare costs.

**TABLE 4:** Effect of chronic conditions on healthcare costs among long‐term survivors of childhood and AYA cancers (*N* = 13,743).

	Un adjusted		*p* > |z|		Adjusted model 1		*p* > |z|		Adjusted model 2		*p* > |z|
	exp (b)	(95% Cl)			exp (b)	(95% CI)			exp (b)	(95% Cl)	
Age at first diagnosis	1.01	(1.00–1.01)	< 0.001	Age at first diagnosis	1.01	(1.00–1.01)	< 0.001	Age at first diagnosis	1.01	(1.00–1.01)	< 0.001
Person years	0.94	(0.93–0.95)	< 0.001	Person years	0.94	(0.93–0.95)	< 0.001	Person years	0.93	(0.92–0.94)	< 0.001
Number of cancers	2.27	(1.75–2.95)	< 0.001	Number of cancers	2.16	(1.74–2.68)	< 0.001				
Chronic condition				Chronic condition				Chronic condition			
Yes	2.9	(2.52–3.25)	< 0.001	Yes	2.3	(2.04–2.59)	< 0.001	Yes	2.3	(2.042–2.60)	< 0.001
Country of birth				Country of birth				Country of birth			
Others^a^	0.86	(0.75–0.98)	0.026	Others	0.88	(0.79–0.99)	0.034	Others	0.85	(0.75–0.96)	0.008
Sex				Sex				Sex			
Female	1.14	(1.03–1.25)	0.01	Female	1.28	(1.29–1.39)	< 0.001	Female	1.28	(1.17–1.38)	< 0.001
Status (31/Dec/2016)				Status (31/Dec/2016)							
Dead	5.46	(4.32–6.91)	< 0.001	Dead	3.74	(3.02–4.62)	< 0.001				
ARIA				ARIA				ARIA			
Regional	0.94	(0.85–1.04)	0.229	Regional	0.97	(0.89–1.06)	0.539	Regional	0.96	(0.87–1.04)	0.401
Remote	0.99	0.73–1.35)	0.972	Remote	0.85	0.66–1.11)	0.306	Remote	0.81	0.62–1.05)	0.116
Unknown	1.01	(0.40–2.56)	0.978	Unknown	0.77	(0.26–2.29)	0.864	Unknown	0.91	(0.29–2.82)	0.866
SEIFA				SEIFA				SEIFA			
Quintile 2	0.84	(0.71–1.00)	0.056	Quintile 2	0.99	(0.85–1.15)	0.915	Quintile 2	0.93	(0.80–1.05)	0.399
Quintile 3	0.91	(0.77–1.08)	0.286	Quintile 3	0.97	(0.84–1.12)	0.7	Quintile 3	0.92	(0.80–1.09)	0.274
Quintile 4	0.9	(0.76–1.06)	0.221	Quintile 4	1.03	(0.90–1.19)	0.658	Quintile 4	0.98	(0.85–1.13)	0.751
Quintile 5	0.89	(0.75–1.06)	0.19	Quintile 5	1.01	(0.87–1.16)	0.916	Quintile 5	0.98	(0.85–1.14)	0.805
Unknown	0.79	(0.40–1.54)	0.486	Unknown	0.72	(0.33–1.58)	0.413	Unknown	0.67	(0.29–1.50)	0.329
Chronic health conditions				Chronic health conditions				Chronic health conditions			
Schizophrenia				Schizophrenia				Schizophrenia			
Yes	3.34	(1.89–5.90)	< 0.001	Yes	1.86	(1.15–3.00)	0.012	Yes	1.64	(1.00–2.71)	0.051
Chronic kidney disease				Chronic kidney disease				Chronic kidney disease			
Yes	7.78	(4.74–12.79)	< 0.001	Yes	3.5	(2.23–5.47)	< 0.001	Yes	3.82	(2.39–6.10)	< 0.001
Heart failure				Heart failure				Heart failure			
Yes	4.45	(2.91–6.80)	< 0.001	Yes	1.38	(0.94–2.02)	0.101	Yes	1.61	(1.07–2.40)	0.021
Paralysis				Paralysis				Paralysis			
Yes	3.35	(2.31–4.84)	< 0.001	Yes	1.34	(0.96–1.83)	0.07	Yes	1.57	(1.13–2.18)	0.007
Epilepsy				Epilepsy				Epilepsy			
Yes	3.46	(2.31–5.19)	< 0.001	Yes	1.31	(0.93–1.86)	0.123	Yes	1.48	(1.03–2.12)	0.033

*Note:* Only long‐term survivors who used at least one of the health services during the assessment period (*N* = 13,743) were included in this analysis.

^a^ Includes other countries besides Australia and whose birthplace wasn't recorded.

Excluding the factors ‘death’ and ‘number of previous cancers’ did not change the relationship between healthcare costs, chronic disease, and other factors (Table 4). Due to the small sample size, being hospitalised with one chronic condition or more had a similar impact on the cost hence the multimorbidity factor was excluded in the final analysis.

## 4 Discussion

We found a notable proportion of long‐term childhood and AYA cancer survivors experienced hospital admissions with a chronic condition as either a primary or secondary diagnosis. Those with chronic conditions had more hospital admissions, longer stays, and higher healthcare costs than survivors without chronic conditions but whom also had hospital admissions. These findings affirm the high burden of chronic illnesses in childhood and AYA cancer survivors reported in previous international studies [9, 32, 33] and demonstrate the substantial health care demand and cost burden associated with cancer in the young population.

International studies, such as those conducted in US and UK [10, 17, 32] report a higher prevalence which likely reflect methodological differences in the studies. For instance, Ehrhardt et al. (2021) conducted a study on chronic conditions in AYA survivors of childhood cancer, using the cumulative burden approach that count multiple reports per condition. The study found an average of 22.3 and 40.3 conditions among those age 18 and 26 years, respectively [10]. Compared to this study's average of one chronic condition per person which was based on number of individual conditions, our method might have produced a conservative estimate of chronic condition prevalence. While multi‐morbidity is common in cancer survivors, few individuals in our cohort exhibited more than one chronic condition. The relatively young survivor population in this study may explain this as multi‐morbidity generally increases with age [34].

Despite our finding of overall annual healthcare costs being lower than those reported in the US and higher than those in France or Taiwan [35, 36] the findings are consistent with previous reports in adult and AYA cancer survivors, showing higher costs for those with chronic conditions [37]. The complex late effects of cancer treatment, combined with ongoing management of chronic conditions drives these costs higher through increased use of additional services like hospitalisations and specialist care [17].

Although conditions such as schizophrenia and chronic kidney disease were relatively uncommon, they were associated with disproportionately high per person costs likely due to the resource intensive hospital‐based treatment procedures such as, dialysis or prolonged psychiatric care. Previous research indicates that compared to other mental health disorders, schizophrenia imposes a disproportionately high economic burden with health care costs nearly twice those associated with effective psychosis [38]. Our study's findings, which are consistent with those previously published by Fitzgerald et al. (2007) further reinforce this impact on the healthcare system [39].Likewise, our cost estimates for chronic kidney disease align with stage‐based variations reported elsewhere [40]. In 2019, people with early‐stage CKD disease experienced costs between AU$41 and AU$2,719, whereas those with stage 4–5 disease or end‐stage renal failure ranged from AU$14,545 to AU$62,000 [40]. Our study's cost estimates fall within this range.

Hypertension and depression were the most common chronic condition in our study similar to reports based on other cohorts of long‐term childhood and AYA cancer survivors [41, 42] which report at least one in four AYA survivors to be affected with mental health disorders, such as depression and over 70% with hypertension [43]. While these conditions are associated with relatively low per person costs, their high prevalence increases the demand of relevant healthcare which cumulatively drives up healthcare costs. Previous studies conducted in New South Wales, Australia [44] and the US, show that cancer survivors had significantly increased use of mental health services, medication and psychotherapy 5 years after diagnosis compared to non‐cancer survivors [41]. Integrating health interventions like psychosocial support, lifestyle modification programs, and targeted mental health interventions in survivorship care may help to reduce the risk of conditions like hypertension and mental health conditions and consequently system‐level costs.

Typically, hospitalisations incur high expenditures compared with other healthcare services as evidenced by literature [35, 36, 45]. Australia's centralised model of paediatric and AYA cancer care, delivered through institutions such Queensland Children's Hospital and Queensland Youth Cancer Service may explain the high inpatient costs and the relatively minimal influence of socioeconomic status and geographic remoteness on healthcare cost variations.

While literature shows end‐of‐life healthcare costs for cancer survivors to be typically high [46], our study excluded the costs associated with the last year of life which implies that the observed high‐cost burden in those who died is likely due the burden of late effects and the chronic survivorship needs rather than terminal care [47].

Survivors with multiple primary cancer diagnoses also showed higher costs which is likely reflective of the cumulative burden of intensive therapies, associated adverse effects and the resulting need for coordinated multidisciplinary care and follow up [48].

Unlike previous studies [46], we did not find a strong relationship between age and healthcare costs. This may reflect our focus on long‐term survivorship (5 years post‐diagnosis) rather than the early post‐treatment period, when age‐related cost variation is more pronounced [46].

### 4.1 Study Limitations

Our study had several strengths and limitations. First, our inability to include services that were sought outside the public healthcare system, such as private hospital and other non‐institutionalised services, underestimated overall health service use and costs. Second, our restriction of the sample to survivors with hospital admissions and the lack of a control group limit direct comparisons with the general population. Lastly, our estimates reflect all‐cause health service use rather than condition specific care and therefore cannot definitively establish the role of specific primary healthcare services in managing chronic conditions.

Balanced against these study limitations, we used data from routinely collected electronic healthcare records, which are void of recall bias, commonly associated with self‐reported responses. In our assessment of chronic condition prevalence, we used simple counts of chronic conditions specified for the ICD‐10 a.m. This approach is supported by Farley et al. [49] who found simple count measures to be better predictors of expenditure when compared to pre‐existing universal comorbidity indices such as the Charlson Comorbidity index [50] and Elixhauser Comorbidity Measure (ECM) [51]. Also, current measures for chronic conditions and comorbidity, which are primarily used to predict mortality and morbidity, lack country‐specific weightings [52]. The large population‐based cohort of our study enhanced representativeness, reduced sampling bias and enabled detection of the less common conditions in the younger age groups. However, given that the last participants in our cohort were diagnosed in 2011, there is a need to update these estimates with more recent data. Since then, new developments in precision medicine and modern treatment guidelines that feature less toxic approaches have further enhanced survival outcomes and potentially reduced the burden of late effects [53]. Furthermore, recent events such as the Covid 19 epidemic could also have had an impact on the long‐term survivorship outcomes and consequently healthcare use patterns and cost trends hence the need for more recent investigations [54].

### 4.2 Clinical Implications

This research presents a unique chronic disease pattern in survivors of childhood and AYA cancer which provides evidence for the Australian National Chronic Condition Management Council and other stakeholders to use in risk evaluation and prevention promotion. In addition, this research supports the move towards value‐based healthcare with the use of payment models that cover full treatment cycles rather than individual services.

Through the optimal care pathways, Queensland cancer treatment and survivorship protocols align with Australian standards [55] which point to the wider implication of our findings beyond Queensland. Although differences in international health systems may limit the extent to which these findings are transferable, they are likely to be reasonably representative of cohorts of young cancer populations in similar high‐income settings.

Future studies need to include data from private hospitals and community‐based providers and indirect costs such as lost productivity to fully understand the economic and societal effects of chronic conditions in survivors of childhood and AYA cancer.

## 5 Conclusion

Our study demonstrates that a sizeable proportion of childhood and AYA cancer survivors are hospitalised due to chronic health conditions including psychological conditions, incurring significantly higher healthcare costs than those without chronic diseases. Implementing holistic survivorship care and preventative measures targeting chronic health conditions in childhood and AYA cancer survivors could optimize healthcare resource use and improve healthcare cost outcomes.

## Author Contributions

**Doreen Nabukalu:** conceptualization, data curation, formal analysis, methodology, writing – original draft, writing – review and editing, project administration. **Louisa G. Collins:** conceptualization, formal analysis, methodology, supervision, validation, writing – review and editing. **Daniel Lindsay:** methodology, writing – review and editing. **John Lowe:** supervision, writing – review and editing. **Katharina M. D. Merollini:** conceptualization, formal analysis, methodology, supervision, validation, writing – review and editing.

## Funding

The authors have nothing to report.

## Conflicts of Interest

The authors declare no conflicts of interest.

## Supporting information


Supporting Information S1

## Data Availability

The data that support the findings of this study are available on request from the corresponding author. The data are not publicly available due to privacy or ethical restrictions.
