# Supplementary material for: Hospitalisations and Costs of Chronic Health Conditions Among Long‐Term Survivors of Childhood, Adolescent, and Young Adult Cancers in Queensland, Australia
Source: Psychooncology. 2026 Feb 7;35(2):e70395. doi: 10.1002/pon.70395 (PMC12882769; doi:10.1002/pon.70395)
Supplement: Supplementary file 1 — Supporting Information S1 [file PON-35-e70395-s001.docx]

**Supplementary tables and figures.**

**Supplementary Figure 1**: Study flow and sample selection


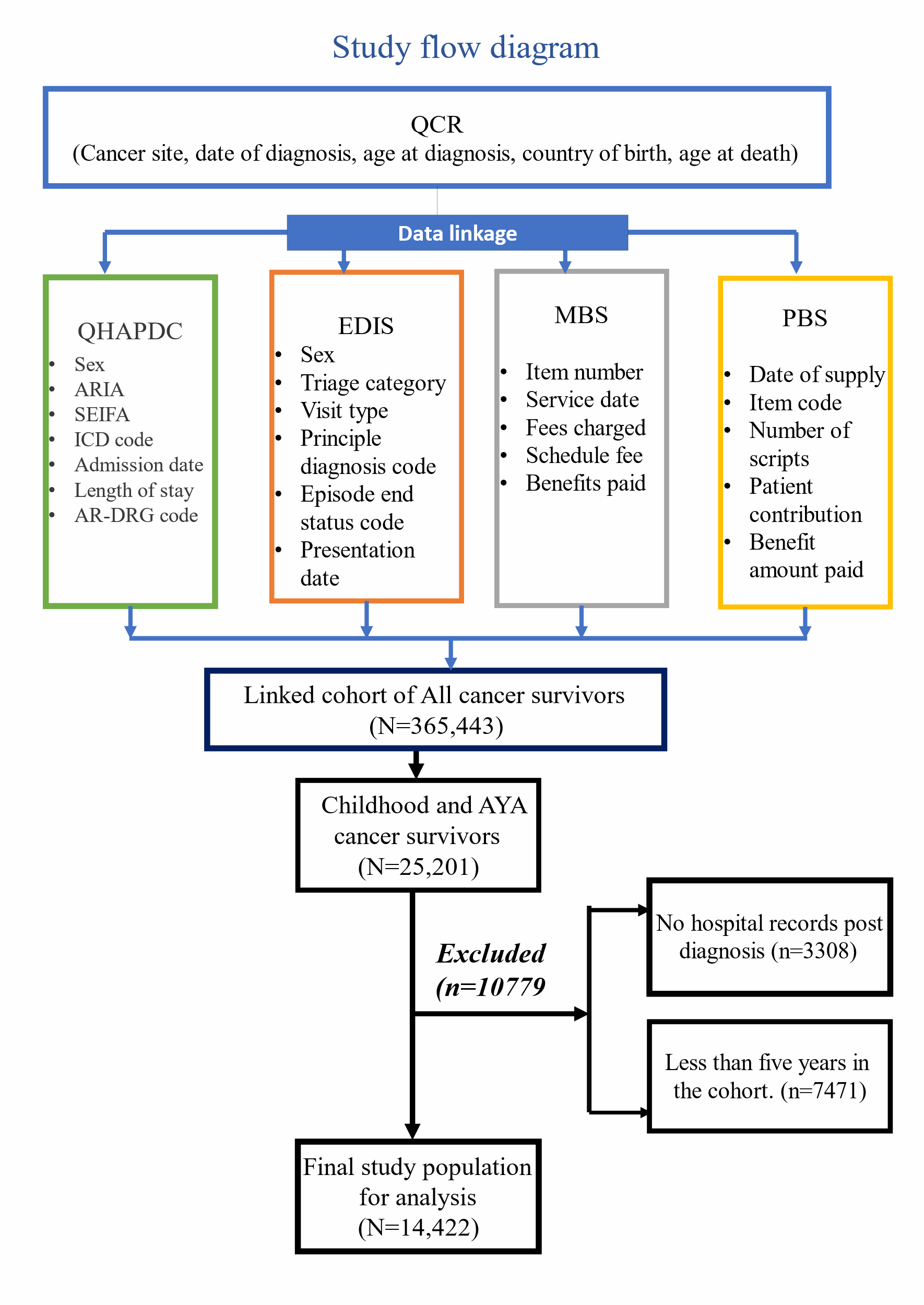


Abbreviations: QCR Queensland Cancer Register; QHAPDC Queensland Hospital Admitted Patient Data Collection; EDIS Emergency department information system, MBS Medicare Benefits Schedule, PBS Pharmaceutical Benefits Scheme, AYA Adolescent and young adults.

**Supplementary Figure 2**: Timing of data analyzed


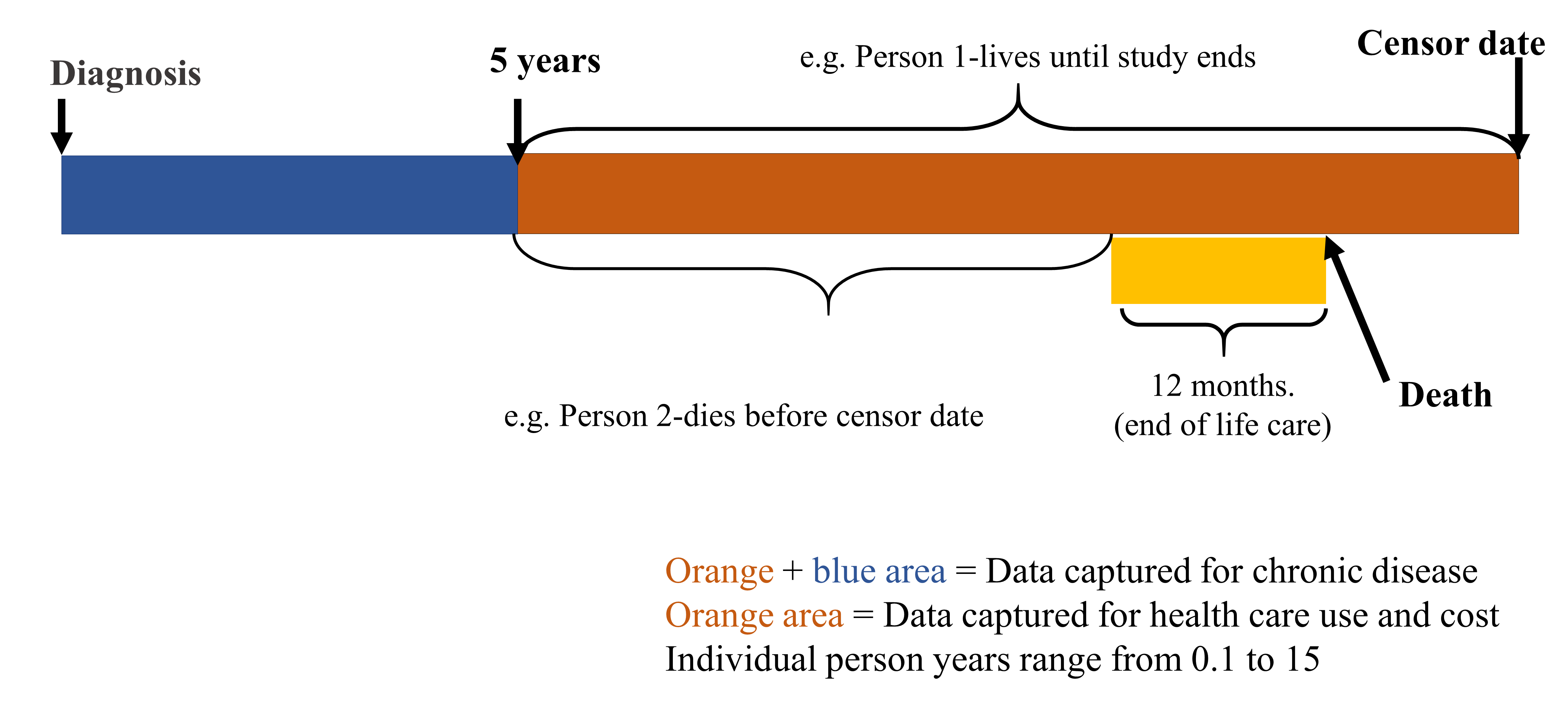


**Supplementary Table 1**: Mean (SD) annual costs per person per year in the long-term phase of care by chronic health condition (N=11554).

| **CONDITION** | N | Mean | SD | p50 | IQR | Sum |
| --- | --- | --- | --- | --- | --- | --- |
| Chronic kidney disease | 25 | $26,428 | $30,331 | $15,888 | ($5,651 - $35,414) | $660,707 |
| Schizophrenia | 44 | $22,835 | $37,204 | $10,460 | ($4,261 - $20,924) | $1,004,753 |
| Epilepsy | 103 | $22,361 | $37,224 | $9,889 | ($4,749 - $18,325) | $2,303,197 |
| Paralysis | 116 | $22,051 | $32,165 | $9,285 | ($4,033 - $27,679) | $2,557,939 |
| Heart failure | 64 | $21,912 | $38,763 | $6,346 | ($2,652 - $20,935) | $1,402,368 |
| Osteoporosis | 15 | $16,885 | $16,759 | $7,185 | ($4,239 - $28,736) | $253,268 |
| Crohn's disease | 57 | $14,995 | $19,451 | $5,350 | ($2,358 - $20,877) | $854,736 |
| Hypertension | 366 | $14,824 | $32,061 | $5,329 | ($1824 - $12,539) | $5,425,562 |
| Depression | 296 | $14,578 | $22,429 | $7,281 | ($3,379 - $15,197) | $4,314,980 |
| Asthma | 186 | $11,383 | $26,310 | $4,422 | ($2,164 - $9,584) | $2,117,251 |
| Others | 357 | $8,098 | $12,369 | $4,795 | ($2,688 - $8,516) | $2,890,860 |
| None | 11554 | $6,798 | $19,055 | $2,294 | ($970 - $5,287) | $78,500,000 |
| *Analysis sample includes long-term survivors with a hospitalisation.* | | | | | | |
